# Cbl-b Enhances Sensitivity to 5-Fluorouracil via EGFR- and Mitochondria-Mediated Pathways in Gastric Cancer Cells

**DOI:** 10.3390/ijms141224399

**Published:** 2013-12-16

**Authors:** Dan Feng, Yanju Ma, Jing Liu, Ling Xu, Ye Zhang, Jinglei Qu, Yunpeng Liu, Xiujuan Qu

**Affiliations:** Department of Medical Oncology, the First Hospital of China Medical University, Shenyang 110001, China; E-Mails: dianafeng1998@gmail.com (D.F.); mayanju2005@gmail.com (Y.M.); cmuliujing@gmail.com (J.L.); cmuxuling@163.com (L.X.); zhangye@mail.cmu.edu.cn (Y.Z.); cmuqujinglei@gmail.com (J.Q.)

**Keywords:** Cbl-b, 5-fluorouracil, EGFR, ERK, PI3k/Akt, gastric cancer

## Abstract

5-Fluorouracil (5-FU) is an essential component of anticancer chemotherapy against gastric cancer. However, the response rate of single drug is still limited. The ubiquitin ligase Cbl-b is a negative regulator of growth factor receptor signaling and is involved in the suppression of cancer cell proliferation. However, whether Cbl-b could affect 5-FU sensitivity remains unclear. The present study showed that Cbl-b knockdown caused higher proliferation concomitant with the decrease of apoptosis induced by 5-FU treatment in gastric cancer cell. Further mechanism investigation demonstrated that Cbl-b knockdown caused significant increase of phosphorylation of EGFR, ERK and Akt, decrease of mitochondrial membrane potential, and increase of expression ratio of Bcl-2/Bax. These results suggest that Cbl-b enhances sensitivity to 5-FU via EGFR- and mitochondria-mediated pathways in gastric cancer cells.

## 1. Introduction

Gastric cancer is one of the most common malignancies worldwide, and is treated mainly with surgery and chemotherapy. In patients with unresectable disease, chemotherapy is the main course of treatment and has been shown to improve overall survival [1]; however, the prognosis remains poor [2]. In addition, adverse effects of cytotoxic chemotherapy often result in deterioration of the quality of life of patients with advanced gastric cancer. Therefore, more effective treatment strategies that are better tolerated are needed.

5-fluorouracil (5-FU) is a widely used chemotherapeutic agent in gastrointestinal malignancies, including gastric cancer [3]. However, the sensitivity of gastric cancer cells to 5-FU is limited; a better understanding of pathways that might contribute to sensitivity or resistance of gastric cancer to 5-FU could therefore contribute towards improvement of the therapeutic effects of 5-FU in gastric cancer. EGFR-dependent survival pathways are known to contribute to chemo-resistance [4] and recent studies suggest the involvement of EGFR-mediated survival signaling in 5-FU sensitivity [5–11]. Thus, mechanisms that regulate EGFR signaling could contribute to 5-FU sensitivity in gastric cancer.

Cbl proteins function as E3 ubiquitin (Ub) ligases [12]. Its RING finger domain recruits aUb-conjugating enzyme (E2); and its tyrosine kinase-binding (TKB) domain (which includes a Src homology 2 domain or SH2 domain) binds to target proteins with cognate phosphotyrosine-containing motifs, which are thereby targeted for Ub modification [13,14]. Cbl-b, a member of the Cbl E3 family, is abundantly expressed in hemopoietic cells and has been demonstrated to negatively regulate the activation of hematopoietic cell lineages such as T cells, B cells, and mast cells via their tyrosine kinase-asscoaited surface receptors [15]. It has also been shown that the p85 regulatory subunit of PI3 kinase (PI3K) serves a substrate for Cbl-b during activation of T cells and Cbl-b functions as a negative regulator of p85 in a ubiquitin-dependent but proteolysis-independent manner [16,17]. Cbl-b-deficient inhibited Akt and ERK phosphorylation and deregulation of T cell proliferation [18]. Our previous studies showed that clinically-used chemotherapeutic drugs, such as etoposide (VP-16), epirubicin (EPI), daunorubicin (DNR), and arsenic trioxide (ATO) upregulated the expression of Cbl proteins in leukemic cell lines and that Cbl-b increased chemosensitivity by inhibiting the PI3K/Akt signaling [19–21]. As the role of Cbl-b in non-hematopoeitic cell systems has not been investigated in detail and activation of EGFR is known to contribute to chemoresistance in epithelial cancers, the present studies examined the relationship of Cbl-b and EGFR in the modulation of tumor cell sensitivity to 5-FU. Our analyses show that Cbl-b depletion through knockdown reduced the chemosensitivity of gastric cancer cells and this was associated with increased proliferation and enhanced EGFR survival signaling, thereby leading us to conclude that induction of Cbl-b promotes chemosensitivity in gastric cancer by restricting the activation of EGFR survival signaling.

## 2. Results and Discussion

### 2.1. Effects of 5-FU on Cell Viability and Apoptosis in Gastric Cancer Cells

**Figure 1.**
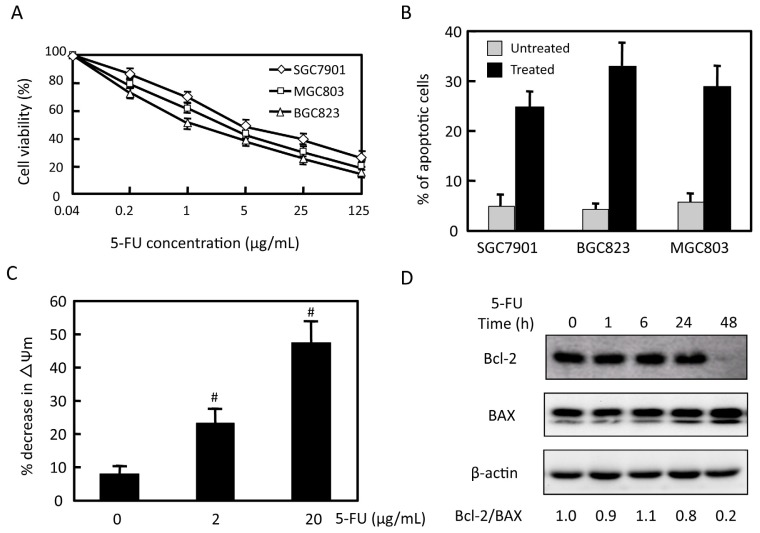
Effects of 5-FU on gastric cancer cell viability and apoptosis. (**A**) Three gastric cancer cell lines (SGC7901, MGC803, and BGC823) were treated with 5-FU for 48 h. Cell viability was tested by the MTT assay. Then a statistical analysis was performed to compare untreated samples and different concentrations of treated samples. The percentage of untreated samples was 100%; (**B**) SGC7901, BGC823, and MGC803 were treated with 2 μg/mL 5-FU for 48 h, apoptosis status was observed by flow cytometry, and results were analyzed by Modifit software; (**C**) MGC803 cells were treated for 48 h with the indicated concentration of 5-FU and mitochondria membrane potential was measured; and (**D**) MGC803 cells were treated with 2 μg/mL 5-FU for the indicated time. Expression of Bcl-2, Bax and β-actin was detected by western blot. Data are expressed as the mean ± SD. ^#^*p* < 0.05. Representative results from at least three individual experiments are shown.

To assess the sensitivity of gastric cancer cells to 5-FU, three gastric cancer cell lines (MGC803, BGC823and SGC7901) were treated with various concentrations of 5-FU for 48 h and reduction in viable cells were assessed using the MTT assay. As shown in Figure 1A, 5-FU triggered dose-dependent cytotoxicity towards all of the cell lines tested with *IC*_50_ values of 2.0 ± 0.9, 1.3 ± 0.8, and 4.5 ± 1.1 μg/mL for MGC803, BGC823and SGC7901 cell lines, respectively. We further assessed the induction by 5-FU in three gastric cancer cell lines after exposure to 2 μg/mL 5-FU for 48 h by measuring a reduction in MTT dye incorporation from baseline (Figure 1B). After 5-FU treatment, 25%, 33% and 29% increase in apoptotic cell was observed in SGC7901, BGC823 and MGC803 cells respectively. To identify the mechanism of 5-FU-induced apoptosis in MGC803 cells, we measured the mitochondrial membrane potential and investigated the levels of proteins related to apoptosis. 5-FU induced decrease of mitochondrial membrane potential in a dose-dependent manner (Figure 1C). Treatment with 2 μg/mL 5-FU for 48 h induced down-regulation of antiapoptotic molecule Bcl-2, and up-regulation of pro-apoptotic molecule Bax. Expression of Bcl-2/Bax ratio decreased 5-fold (Figure 1D). These results indicate that 5-FU inhibits proliferation and promotes apoptosis in gastric cancer cells.

### 2.2. Effects of 5-FU on EGFR, ERK, and Akt Expression in MGC803 Cells

**Figure 2.**
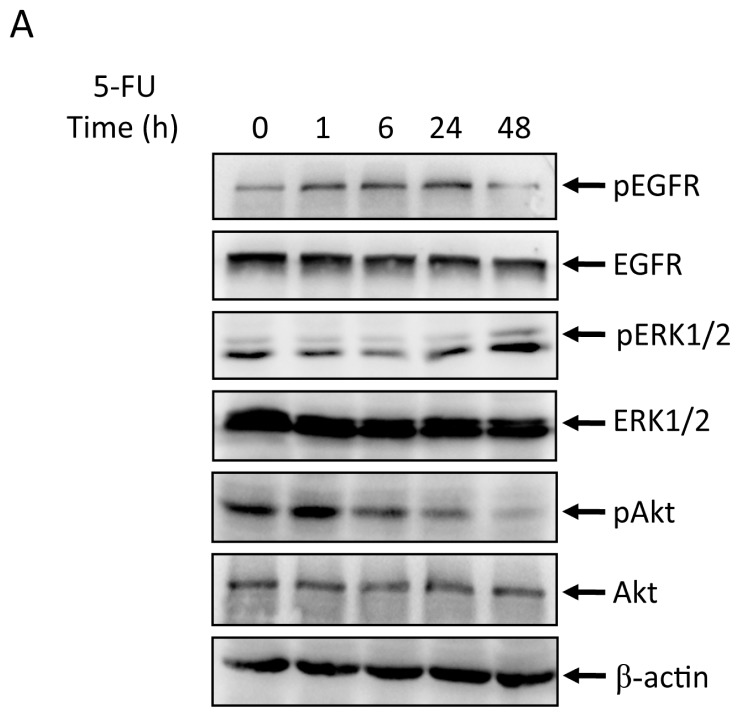

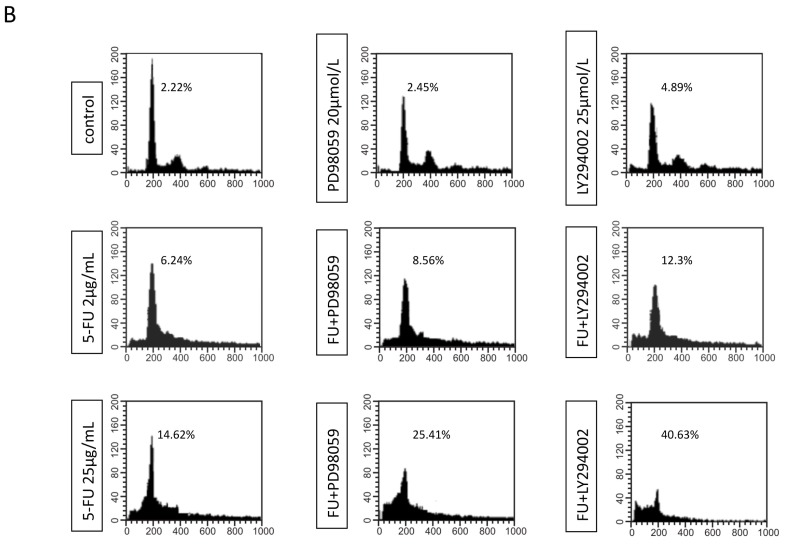
Effects of 5-FU on pEGFR, EGFR, pERK, ERK, pAkt and Akt expression in MGC803 cells. MGC803 cells were treated with 2 μg/mL 5-FU for the indicated time. (**A**) pEGFR, EGFR, pERK, ERK, pAkt, and Akt expression was evaluated by western blot; and (**B**) MGC803 cells were treated with 2 μg/mL 5-FU in the absence or presence of MAPK inhibitor of PD98059 or PI3K inhibitors of LY294002. Then the percentage of apoptotic cells was analyzed by flow cytometry. Representative results from at least three individual experiments are shown.

The effects of 5-FU (2 μg/mL) on phosphorylation of EGFR, ERK, and Akt in MGC803 cell line were tested by western blot analysis. The levels of phospho-EGFR were elevated by 1 h, peaked at 6 h and decreased by 48 h. Phospho-ERK and phospho-Akt levels also increased (Figures 2A). We next investigated whether inhibitors of MAPK (PD98059) or PI3K (LY294002) could affect 5-FU-induced apoptosis in MGC803 cells. As shown in Figure 2B, 20 μmol/L PD98059 or 25 μmol/L LY294002 alone had little effect on apoptosis. However, when cells were pretreated with PD98059 or LY294002 and then exposed to 25 μg/mL 5-FU, the percentage of apoptotic cells increased from 14.62% to 25.41% and 40.63%, respectively. These results are consistent with a role for the EGFR/ERK/Akt signaling pathway in modulating the level of 5-FU induced apoptosis in MGC803 gastric cancer cells.

### 2.3. Effects of EGFR Inhibitor and 5-FU on ERK and Akt in MGC803 Cells

**Figure 3.**
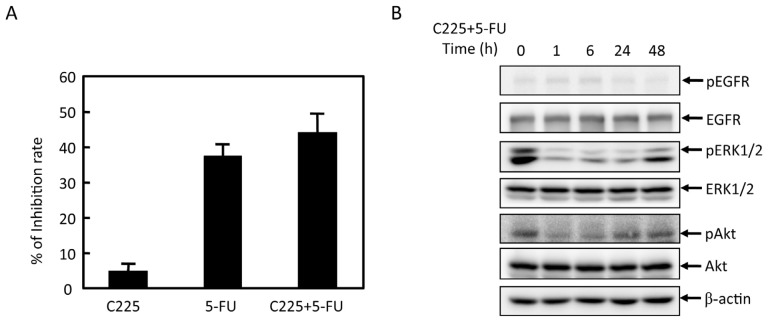
Effects of EGFR inhibitor (C225) on pERK, ERK, pAkt and Akt expression upon 5-FU treatmentin MGC803 cells. (**A)** MGC803 cells were pretreated with 10 μg/mL C225, and then treated with 5-FU for the indicated time. Inhibition rate was tested by MTT assay. The percentage of untreated samples was 100%; and (**B**) pEGFR, EGFR, pERK, ERK, pAkt, and Akt expression was evaluated by western blot. Representative results from at least three individual experiments are shown.

We examined the effects of EGFR inhibitor (C225) and 5-FU on MGC803 cells proliferation. As shown in Figure 3A, with combined treatment of 5-FU and C225, proliferation inhibition rate increased from 37% to 44%. As shown in Figure 3B, p-EGFR, pERK and pAkt expression decreased. These results indicated that C225 and 5-FU showed additional antitumor effect. Combined treatment of 5-FU and C225 significantly inhibit the activation of Akt and ERK.

### 2.4. Effects of Cbl-b on 5-FU Chemosensitivity in MGC803 Cells

**Figure 4.**
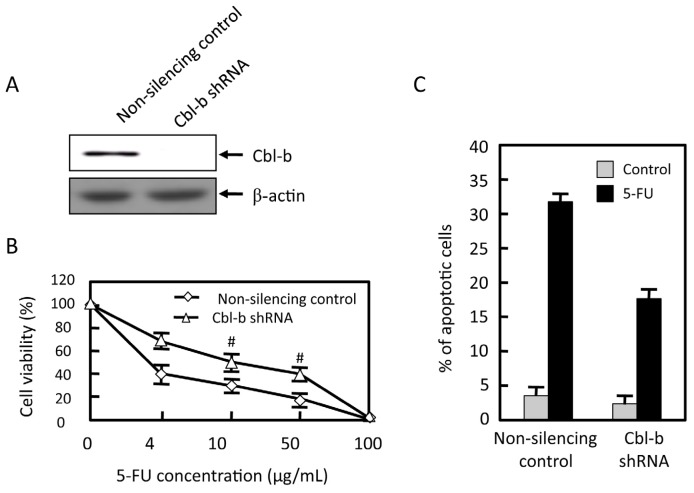

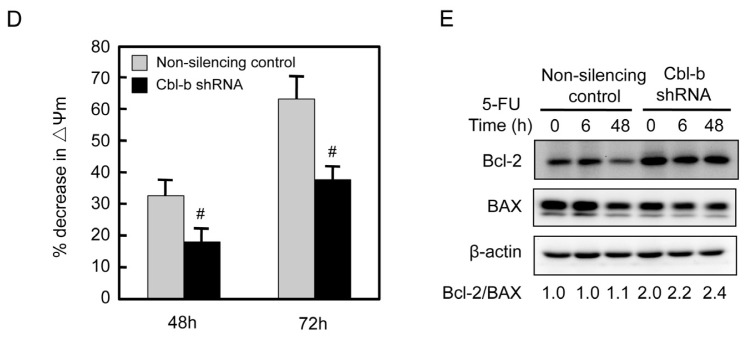
Effects of Cbl-b on 5-FU chemosensitivity. MGC803 cells were transfected by non-silencing control and Cbl-b shRNA. (**A**) Cbl-b and β-actinexpression were evaluated by western blot; (**B**) Then cells were treated with the indicated concentration of 5-FU for 48 h, and cell viability tested by MTT assay. Statistical analysis was performed. The percentage of untreated samples is 100%; (**C**) MGC803 cells were transfected by non-silencing control and shRNA for Cbl-b, followed by treatment with 2 μg/mL 5-FU for 48 h. Apoptotic cells were observed by flow cytometry, and final results were analyzed with Modifit software; (**D**) The cells were treated with 2 μg/mL 5-FU for 48 and 72 h and mitochondria membrane potential was measured; and (**E**) The cells were treated with 2 μg/mL 5-FU for 6 and 48 h. Expression of Bcl-2, Bax and β-actin were detected by western blot. ^#^*p* < 0.05. Representative results from at least three individual experiments are shown.

We previously established stable cell line of non-silencing control shRNA and Cbl-b shRNA in MGC803 cells as a tool to examine the mechanism of Cbl-b. As shown in Figure 3A, Cbl-b expression was essentially undetectable in Cbl-b shRNA of MGC803 cells, whereas it was detected in non-silencing control of MGC803 cells (Figure 4A). Notably, analyses using the MTT assay (Figure 4B) showed that knockdown of Cbl-b led to relative resistance to 5-FU: compared to an *IC*_50_ value of 2.57 ± 0.7 μg/mL for non-silencing control cells, the *IC*_50_ value for Cbl-b knockdown cells was 10.67 ± 5.0 μg/mL (*p* = 0.001). Furthermore, flow cytometric analysis of apoptosis showed reduced level of apoptosis in Cbl-b shRNA cells: 18% ± 3.0% apoptotic cells were observed in Cbl-b shRNA cells treated with 2 μg/mL 5-FU for 48 h compared to 32% ± 4.0% apoptotic cells in the non-silencing control cell line (Figure 4C; *p* = 0.001). 5-FU induced the increase of mitochondrialmembrane potential was reversed by Cbl-b knockdown (Figure 4D). Expression of Bcl-2/Bax ratio was also increased (Figure 4E). Knockdown of Cbl-b promotes the proliferation of gastric cancer cells and inhibits their apoptosis. These results indicate that Cbl-b enhances sensitivity to 5-fluorouracil via mitochondria-mediated pathways in gastric cancer cells.

### 2.5. Effects of Cbl-b on 5-FU-Induced EGFR, REK, and Akt Activation

**Figure 5.**
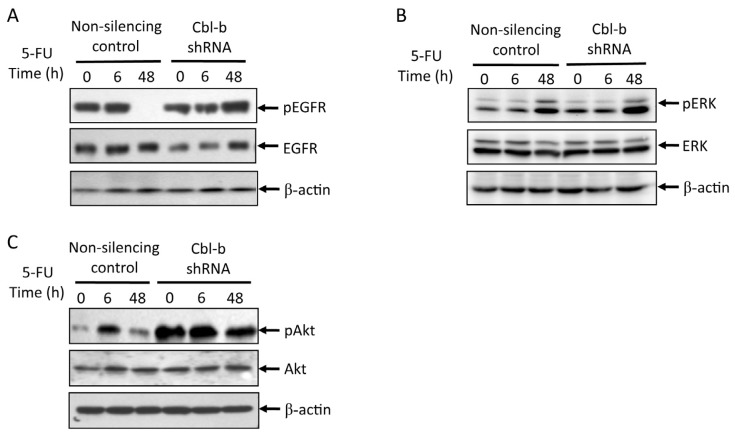
Effects of 5-FU on pEGFR, EGFR, pERK, ERK, and pAkt and Akt expression in MGC803 cells transfected with non-silencing control and Cbl-b shRNA. (**A**–**C**) MGC803 cells were treated by 2 μg/mL 5-FU for 0, 6, and 48 h. pEGFR/EGFR, pERK/ERK, and pAkt/Akt expression was tested by western blot. Representative results from at least three individual experiments are shown.

Since Cbl proteins are negative regulators of EGFR signaling, Cbl-b may promote 5-FU chemosensitivity in gastric cancer cells by regulating the level of EGFR survival signaling. To evaluate this hypothesis, we compared the levels of phosphorylated EGFR, ERK, and Akt activation upon 5-FU treatment in non-silencing control *vs.* Cbl-b shRNA expressing MGC803 cells. Western blot analyses of lysates from cells treated with 2 μg/mL 5-FU for 6 and 48 h (Figure 5A) showed that while phosphorylation of EGFR diminished to almost undetectable levels by 48 h in control vector-expressing cells, the signals were still very strong in Cbl-b shRNA cells. Sustained signals were also observed for pERK and pAkt in Cbl-b knockdown cells compared to control cells (Figure 5B,C). These results support the proposal that Cbl-b promotes chemosensitivity of gastric cancer cells by limiting EGFR survival signaling via ERK and Akt.

## 3. Experimental Section

### 3.1. Reagents and Antibodies

3-(4,5-Dimethylthiazol-2-yl)-2,5-diphenyltetrazolium bromide (MTT) and dimethylsulphoxide (DMSO), PD98059 and LY294002 were from Sigma-Aldrich (St. Louis, MO, USA). 5-Fluorouracil (5-FU) was obtained from Wako Inc. (Wako Chemicals, Richmond, VA, USA). C225 were from Merck (Darmstadt, Germany). Antibodies against Cbl-b, Bcl-2, Bax and β-actin were from Santa Cruz Biotechnology (Santa Cruz, CA, USA). Antibodies against EGFR and phospho-EGFR, ERK and phospho-ERK, Akt and phospho-Akt were from Cell Signaling Inc. (Frankfurt am Maine, Germany).

### 3.2. Cells and Cell Culture

The gastric cancer cell lines (MGC 803, BGC 823 and SGC 7901) were obtained from the Type Culture Collection of the Chinese Academy of Sciences (Shanghai, China). The cells were cultured in RPMI-1640 medium (GIBCO, Gaithersburg, MD, USA) containing 10% fetal bovine serum (FBS), penicillin (100 U/mL), and streptomycin (100 mg/mL) at 37 °C in an atmosphere of 95% air and 5% CO_2_.

### 3.3. RNA Interference Stable Infection

Sense and antisense oligonucleotides (Human Cbl-b sepcific sequence: 5′-GATCCCGTTTCCGGTTAAGTTGCACTCGTTCAAGAGACGAGTGCAACTTAACCGGAAATTTTTTCCAAA-3′ and 5′-AGCTTTTGGAAAAAATTTCCGGTTAAGTTGCACTCGTCTCTTGAACGAGTGCAACTTAACCGGAAAGG-3′ for Cbl-b; Non-silencing control: 5′-GATCCCGTTCTCCGAACGTGTCACGTTTGATATCCGACGTGACACGTTCGGAGAATTTTTTCCAAA-3′ and 5′-AGCTTTTGGAAAAAATTCTCCGAACGTGTCACGTCGGATATCZAACGTGACACGTTCGGAGAACGG-3′) were phosphorylated with T4 kinase (Takara, Tokyo, Japan), annealed, and ligated into BamHI/HindIII-cleaved pRNA-U6.1/Neo vector (Genscript, Piscataway, NJ, USA). shRNA-expressing plasmids were transfected into MGC803 cells using the Lipofectamine 2000 reagent (Invitrogen, Carlsbad, CA, USA). After 48 h, the medium was supplemented with 0.6 mg/mL G418 (Life Technologies, Carlsbad, CA, USA) for selection of stable transfectants (for 10 days) followed by serial passage in the same medium.

### 3.4. MTT Assay

The effects of various agents on cell proliferation were measured using the MTT assay. Cells were seeded at 1 × 10^4^/well in 96-well plates and incubated overnight; various concentrations of test agents were then added and incubation continued for 48 h. Thereafter, 20 μL of MTT solution (5 mg/mL) was added to each well and the cells incubated for a further 4 h at 37 °C. After removal of the culture medium, cells were lysed in 200 μL of DMSO and the optical density (OD) was measured at 570 nm using a microplate reader (Bio-Rad, Hercules, CA, USA). Change in percentage viability was calculated using the formula: OD of the experimental sample/OD of the control group × 100%.

### 3.5. Flow Cytometry Analysis

The cells were seeded at 3 × 10^5^ cells/well in six-well plates for 24 h and further cultured in the presence or absence of 5-FU for the indicated time. Cells were collected and washed twice with PBS and fixed in ice-cold 70% ethanol for 12 h. The samples were then washed twice with PBS and incubated with 20 μg/mL RNase A and 10 μg/mL propidium iodide (PI) at room temperature in dark for 30 min, and DNA content was determined by flow cytometry using a FACScan flow cytometer (Becton Dickinson, San Jose, CA, USA), and data were analyzed using Modifit software (Becton Dickinson, San Jose, CA, USA). The apoptotic rate was determined on the basis of the “sub-G1” peak.

### 3.6. Measurement of *Mitochondrial* Membrane Potential

For determination of mitochondrial transmembrane potential (ΔΨ_m_), cells were incubated with 3,3′-dihexyloxacarbocyanine iodide (DiOC6) in culture media at 37 °C for 15 min, then analyzed with a FACScan flow cytometer (Becton Dickinson, San Jose, CA, USA).

### 3.7. Immunoblotting

Cells were washed twice with ice-cold PBS and solubilized in 1% Triton lysis buffer (1% Triton X-100, Sigma, St. Louis, MO, USA, 50 mM Tris, pH 7.4, 150 mM NaCl, 10 mM EDTA (ethylene diaminete traacetic acid), 100 mM NaF, 1 mM Na_3_VO_4_, 1 mM phenylmethyl sulfonyl fluoride, and 90 mU/mL aprotinin) on ice. For the preparation of total cell lysates, cells were washed as described earlier with pre-chilled PBS and lysed with 1% Triton lysis buffer, followed by addition of 3× sample buffer and heating at 100 °C for 5 min. Cell lysates were separated by SDS-polyacrylamide gel electrophoresis (Tanon Science & Technology Co., Ltd., Shanghai, China) and electrophoretically transferred to polyvinylidenedifluoride transfer membrane (Millipore, Bedford, MA, USA). After blocking with 5% skim milk in Tris Buffered Saline with Tween 20 (10 mM Tris, pH 7.4, 150 mM NaCl, and 0.1% Tween 20), the blots were probed with the indicated primary antibodies for 1 h at 4 °C, followed by the appropriate horseradish peroxidase-conjugated secondary antibody for 30 min at room temperature. Finally, proteins were visualized using enhanced chemiluminescence reagents (SuperSignal Western Pico Chemiluminescent Substrate; Pierce, Rockford, IL, USA). The signals on blots were analyzed using the NIH Image J software (http://rsb.info.nih.gov/ij/).

### 3.8. Statistical Analysis

Where appropriate, differences were analyzed for statistical significance using Student’s *t*-test. *p* < 0.05 was considered statistically significant. The results are expressed as means ± SD.

## 4. Conclusions

5-Fluorouracil (5-FU) has been extensively used in the treatment of gastrointestinal, pancreatic, and breast cancers. Studies in human pancreatic carcinoma cells have revealed that 5-FU induces EGFR and Akt phosphorylation [22]. As PI3K/Akt signaling pathway promotes survival, these findings have led to the suggestion of a role for induced EGFR/ERK/Akt pathway in reducing chemosensitivity [4,23–25]. The mechanisms of chemoresistance to 5-FU in gastric cancer are however less clear. Studies presented here support a role for Cbl-b E3 ubiquitin ligase in promoting chemosensitivity of gastric cancer cells to 5-FU through a mechanism that involves Cbl-b-dependent limitation of ERK and Akt survival signaling downstream of EGFR.

Our studies demonstrated that 5-FU triggered dose-dependent cytotoxicity in three gastric cancer cell lines (SGC7901, MGC803, and BGC823), and induced apoptosis in a time-dependent manner. Western blot analyses indicated that after 5-FU treatment, the levels of phospho-EGFR and phospho-Akt elevated in a short time and finally decreased at 48 h. The levels of phospho-ERK transiently decreased and then elevated by 48 h in MGC803 cells, which implied that the EGFR/ERK/Akt signaling pathway may be involved in 5-FU activity in gastric cancer cells. Knockdown studies of Cbl-b directly showed that loss of Cbl-b led to chemoresistance to 5-FU, as well as enhanced activation of EGFR, ERK and Akt signaling. These results indicated that Cbl-b enhanced the chemosensitivity of gastric cancer cells to 5-FU through the regulation of levels of active (phosphorylated) EGFR, ERK and Akt.

Our suggestion that Cbl-b functions together with EGFR/ERK/Akt pathway regulating the balance between chemosensitivity and chemoresistance to 5-FU is supported by findings in the literature. In related studies, we have shown that the chemotherapeutic agent, oxaliplatin, can sensitize gastric cancer cells to TRAIL by regulating components of the apototic/survival machinery such as caspase-3, caspase-8, Bax, and Bcl-2 protein expression [26]. Cbl proteins are known to function as E3-ubiquitin ligases and restrict receptor tyrosine kinase (RTK) signaling, most notably EGFR [27–30]. While Cbl-b function has been predominantly studied in the context of hematopoietic cells, its ability to negatively regulate EGFR signaling has been well documented in model cell systems. For example, Ettenberg demonstrated that Cbl-b was phosphorylated and recruited to EGFR upon EGF stimulation, and was found to bind to the Grb2 adaptor protein. Furthermore, 32D/EGFR cells overexpressing Cbl-b showed markedly reduced proliferative response to EGF, and increased the number of cells undergoing apoptosis [31]. Ahsan reported that Cbl-dependent EGFR degradation might contribute to sensitivity of head and neck cancer cells to cisplatin [32]. Our previous results also showed that an involvement of Cbl-b in apoptosis induced by anthracyclines in gastric cancer cells [19]. Together, these previous studies further support an involvement of Cbl-b and its potential regulation of EGFR signaling in determining chemosensitivity of gastric cancer cells. The knockdown studies of Cbl-b and biochemical analyses of EGFR signaling in the present study directly support this idea. Notably, while the role of Cbl proteins as physiological negative regulators of tyrosine kinases is now well-established, modulation of these ubiquitin ligases to regulate the balance between pro-survival and apoptotic signaling during anticancer therapy is an emerging area with substantial clinical implications. Thus, insights gained here should be helpful in the design of further studies to identify ways to enhance the effectiveness of chemotherapy by modulating Cbl protein-dependent degradation of tyrosine kinases.
